# Molecular Characteristics of Subgenomic RNAs and the Cap-Dependent Translational Advantage Relative to Corresponding Genomic RNAs of *Tomato spotted wilt virus*

**DOI:** 10.3390/ijms232315074

**Published:** 2022-12-01

**Authors:** Chen Yang, Chengming Yu, Zhenjia Zhang, Deya Wang, Xuefeng Yuan

**Affiliations:** Shandong Province Key Laboratory of Agricultural Microbiology, Department of Plant Pathology, College of Plant Protection, Shandong Agricultural University, Tai’an 271018, China

**Keywords:** *tomato spotted wilt virus* (TSWV), subgenomic RNAs (sgRNAs), cap-dependent translation, ambisense RNA virus

## Abstract

*Tomato spotted wilt virus* (TSWV) causes severe viral diseases on many economically important plants of Solanaceae. During the infection process of TSWV, a series of 3′-truncated subgenomic RNAs (sgRNAs) relative to corresponding genomic RNAs were synthesized, which were responsible for the expression of some viral proteins. However, corresponding genomic RNAs (gRNAs) seem to possess the basic elements for expression of these viral proteins. In this study, molecular characteristics of sgRNAs superior to genomic RNAs in viral protein expression were identified. The 3′ ends of sgRNAs do not cover the entire intergenic region (IGR) of TSWV genomic RNAs and contain the remarkable A-rich characteristics. In addition, the 3′ terminal nucleotides of sgRNAs are conserved among different TSWV isolates. Based on the eIF4E recruitment assay and subsequent northern blot, it is suggested that the TSWV sgRNA, but not gRNA, is capped in vivo; this is why sgRNA is competent for protein expression relative to gRNA. In addition, the 5′ and 3′ untranslated region (UTR) of *sgRNA-Ns* can synergistically enhance cap-dependent translation. This study further enriched the understanding of sgRNAs of ambisense RNA viruses.

## 1. Introduction

The accelerated life cycle of viruses requires efficient and accurate translation of virally encoded proteins [1,2]. Several families of RNA viruses have the ability to encode multiple genes per single genomic RNA molecule through the production of subgenomic mRNAs (sgRNAs) during the infection cycle [3,4,5]. These sgRNAs generally have 5′ ends identical to the genomic RNA and 3′ ends featuring deletions [6,7]. Examples of viral plant pathogens which produce sgRNAs include *tobacco mosaic virus* (TMV) and *barley yellow dwarf virus* (BYDV) [7,8,9,10,11].

While the mechanism and regulation of sgRNA synthesis are well characterized in these and related viruses, less is known regarding sgRNA translation. Typically, cellular mRNAs possess two codependent translational regulators, a 5′m7GpppX cap and a poly(A) tail, which interact synergistically to promote interaction between mRNA termini [12,13,14]. The 5-m7GpppN cap structure has multiple roles in gene expression, including enhancement of RNA stability, splicing, and translation initiation. However, many plant-specific RNA viruses lack a cap and/or a poly(A) tail, and instead have evolved cap-independent translation strategies to compete with host translation [12,14,15,16]. To date, two types of cap-independent translational elements have been characterized, namely, the internal ribosome entry site (IRES) located in the 5′ terminal region, and the 3′cap-independent translation element (3′CITE) located in the 3′ UTR [16,17].

*Tomato spotted wilt virus* (TSWV), belonging to the genus Tospovirus of the family Bunyaviridae, is one of the top ten important plant viruses causing significant economic damage to several vegetables and ornamental plants worldwide [18,19,20]. The TSWV genome consists of three single-stranded RNAs. The large (*L*) RNA is in negative sense, while the middle (*M*) and small (*S*) RNAs are ambisense and contain two non-overlapping genes on opposites strands, separated by a large internal gene region (IGR) [21,22,23,24]. The *L* RNA encodes an RNA-dependent RNA polymerase (RdRp). The *M* RNA encodes precursors of two structural glycoproteins (GN and GC), and one non-structural protein (NSm). The *S* RNA encodes the nucleocapsid protein (N) and one non-structural protein (NSs) [19,25,26]. The genes encoded by the TSWV ambisense RNA fragments are expressed through the synthesis of sgRNAs and their transcription is initiated by the ‘cap snatching’ mechanism, which was first identified in TSWV [22,27,28].

During the process of ‘cap snatching’, the viral RNA polymerase cleaves a 7mG-capped RNA leader from a host mRNA for use as a primer for viral genomic transcription [29,30,31]. As a result, the viral RNAs gain a 5′ cap structure, and this is likely critical for translation initiation. It is unclear why TSWV viral protein biosynthesis relies on sgRNA as a translation template despite the fact that TSWV gRNAs encode proteins. Previous studies have revealed that the TSWV *S*-RNA-derived mRNA transcription termination signal is located near the 3′ end of the intergenic hairpin structure, which acts to enhance translational efficiency [32,33]. However, whether the TSWV gRNAs and sgRNAs are capped in vivo, and what role the TSWV sgRNA UTRs play in translational regulation, are still open questions.

Here, we sought to more thoroughly investigate the translational regulation of TSWV proteins. Specifically, we determined the precise location of the TSWV sgRNA 3′ terminal region, the cap status of the TSWV gRNAs and sgRNAs, and the cis-acting elements regulating TSWV sgRNA translation. We found that the TSWV *sgRNA-NSs* is capped in vivo, but not the *S* RNA, and that both the sgRNA 5′ and 3′ UTRs play an important role in cap-dependent translation. Specifically, TSWV sgRNA translation is coordinately enhanced by both the 5′ and 3′ UTRs in a cap-dependent manner.

## 2. Results

### 2.1. Mapping of 3′ Terminal Nucleotide in the TSWV sgRNAs

**Figure 1:**
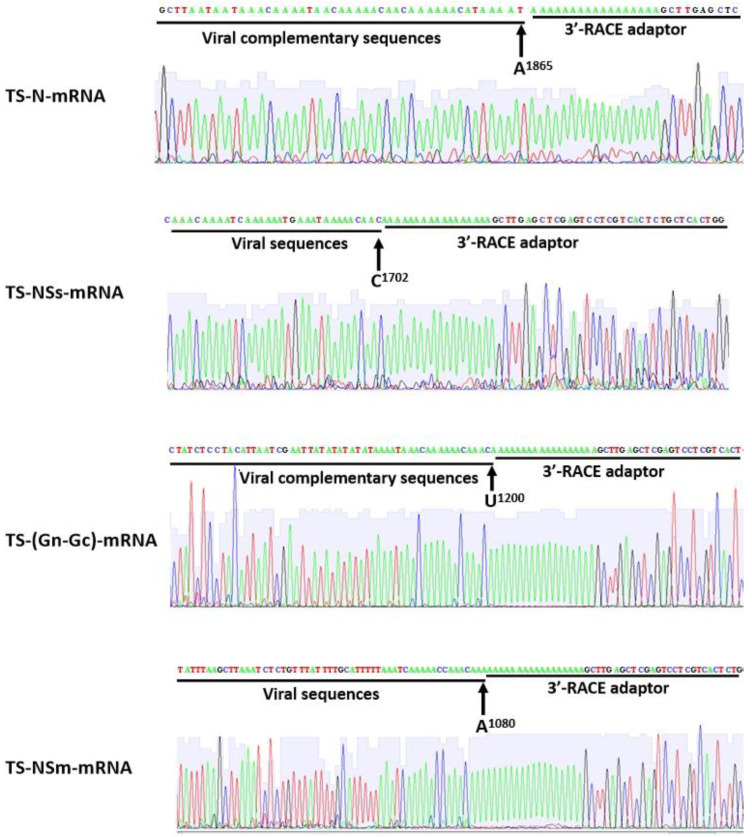
Precise mapping of TSWV sgRNA 3′ terminal region nucleotide sequences by 3′RACE. Note: The locations marked by numbers represent viral strands.

Previous studies indicated that the transcription termination site of the *S* RNA-encoded *N* mRNA is located at the 1568–1574 position, and for *NSs* mRNA is at the 1852–1839 position. To ascertain the precise positioning of the 3′ terminal nucleotides of the TSWV *M* (KM65718)- and *S* (KM657115)-*M*-encoded sgRNAs, 3′RACE was carried out. Our results suggest that the 3′ terminal nucleotide of *sgRNA-NSm* (5′ co-terminal with RNA *M*) was A1080 in RNA *M*, while the 3′ terminal nucleotide of *sgRNA-(Gn-Gc)* (5′ co-terminal with the complementary strand of RNA *M*) was complementary to U1200 in RNA *M* (Figure 1). The 3′ terminal nucleotide of sgRNA-*NSs* (5′ co-terminal with RNA *S*) was C1702 in RNA *S*, while the 3′ terminal nucleotide of sgRNA-*N* (5′ co-terminal with the complementary strand of RNA *S*) was complementary to A1865 in RNA *S* (Figure 1).

### 2.2. The 3′ End of TSWV sgRNAs Located at the Side of the A-Rich Region of IGR

**Figure 2:**
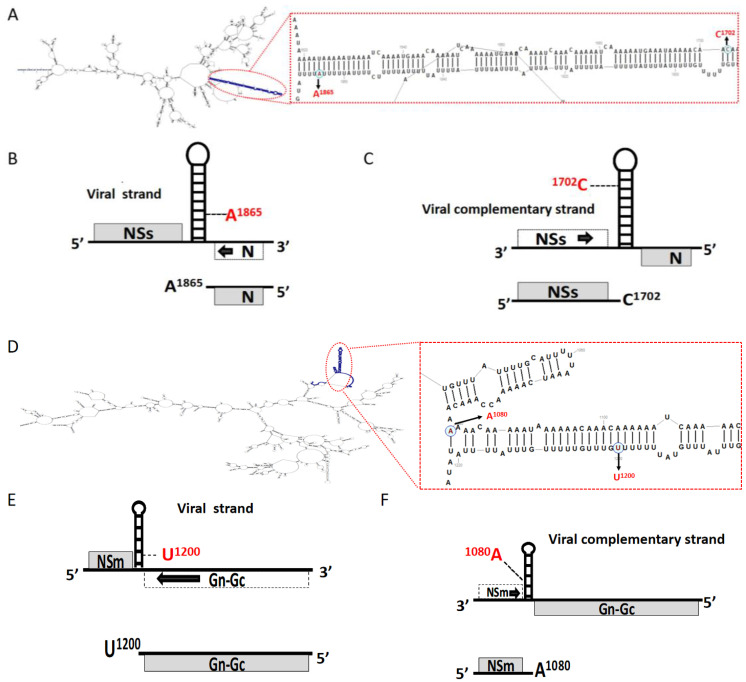
Location of 3′ terminal nucleotide of sgRNAs within the intergenic regions (IGR) of RNA *M* and RNA *S* as well as their compliment strands. (**A**) Predicted secondary structure of TSWV RNA *S* genome, broken circle indicating the stem-loop in the IGR. (**B**) Organization of *sgRNA-N* and location of 3′ terminal nucleotide on the stem-loop. (**C**) Organization of *sgRNA-NSs* and location of 3′ terminal nucleotide on the stem-loop. (**D**) Predicted secondary structure of TSWV RNA *M* genome, broken circle indicating the stem-loop in the IGR (**E**). Organization of *sgRNA-(Gn-Gc)* and location of 3′ terminal nucleotide on the stem-loop. (**F**) Organization of *sgRNA-NSm* and location of 3′ terminal nucleotide on the stem-loop.

The TSWV ambisense *M*- and *S*-RNA segments contain an A/U-rich intergenic region (IGR), which is predicted to form a stable hairpin structure (Figure 2A,D), and TSWV transcription is reported to terminate near the 3′ end of this hairpin structure, and consequently contain the entire stem-loop structure. However, our predicted TSWV RNA (S and M) IGR secondary structure indicated that the 3′ terminal nucleotide of all sgRNAs (*N*, *NSs*, *NSm*, and *Gn-Gc*) do not cross the top of the intergenic hairpin (Figure 2B,C,E,F), and instead disrupt the base pairing between A and G. Furthermore, the 3′ UTRs of TSWV sgRNAs contained only partial stem-loop structures within the viral or complementary RNA *M* and *S* strands, leading to single-stranded A-rich regions at the 3′ terminal region of each sgRNA.

### 2.3. Conservation Analysis of TSWV RNA M and S IGRs

**Figure 3:**
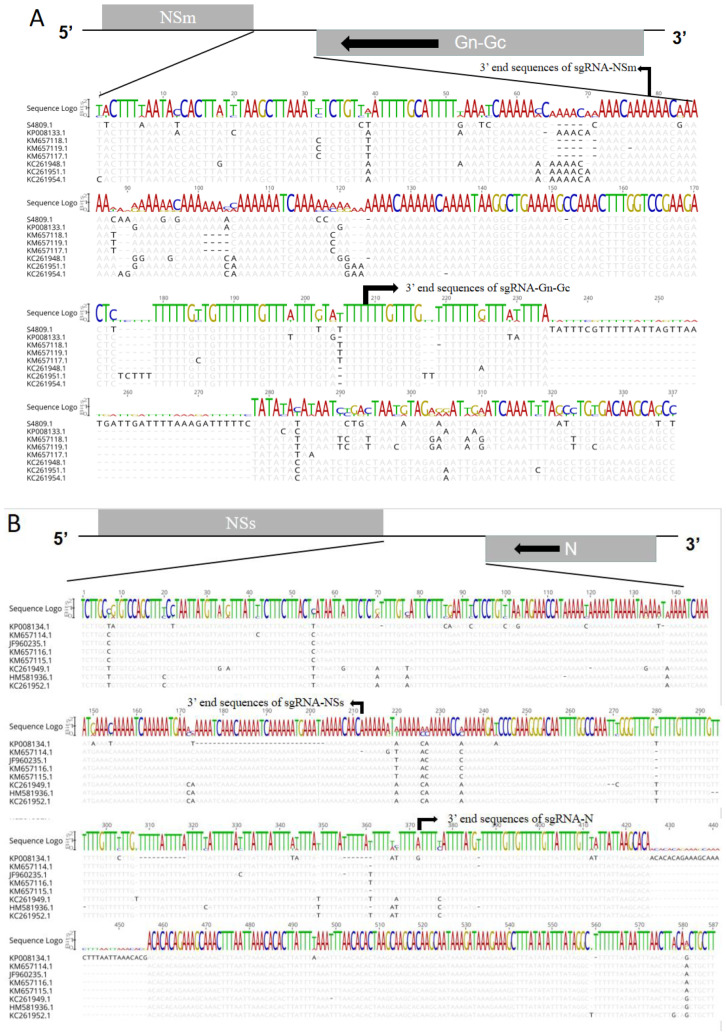
TSWV RNA *M* and *S* intergenic region (IGR) alignment. (**A**) Alignment of the nucleic acid sequences of IGR of RNA *M*. (**B**) Alignment of the nucleic acid sequences of IGR of RNA *S*.

Sequence analysis indicated that TSWV RNA *M* and *S* were highly variable and ranged in length from 251 to 340 nt, largely due to differences in IGR sequence length. To examine sgRNA 3′ UTR conservation, we compared our cloned IGR sequences with 8 IGR sequences reported in the NCBI database. Overall, although the length and sequences of IGRs across the examined TSWV RNA *M* and *S* differed, all exhibited conserved sgRNA 3′ terminal regions and flanking sequences. The TSWV RNA *M* exhibited highly conserved 3′ end and upstream sequences in both sgRNA-NSm and *sgRNA-(Gn-Gc)*, including the consensus sequences AAACAAA1080 and U1200GUUU(G/A)UU, respectively (Figure 3A). The TSWV RNA *S* also exhibited highly conserved 3′ end and upstream sequences in both *sgRNA-NSs* and *sgRNA-N*, including the consensus sequences (A/G)A(C/U)AAC1702 and A1865UUUU(A/U)UGUUUU, respectively (Figure 3B).

### 2.4. Effect of 5′ and 3′ UTR of TSWV gRNA and sgRNA on Cap-Dependent or Cap-Independent Translation

**Figure 4:**
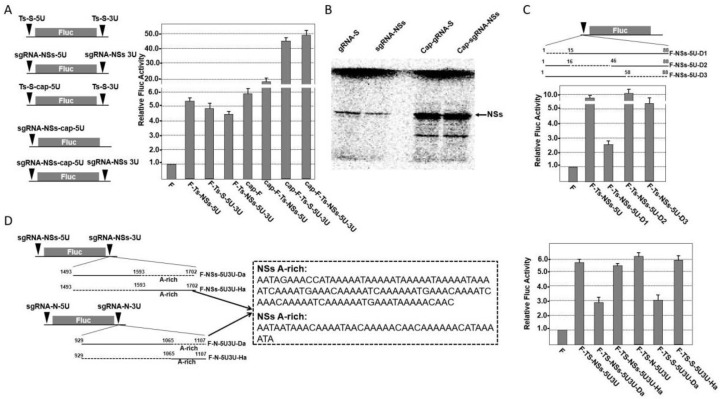
Translational characteristics of TSWV RNA *S* and *sgRNA-NSs*. (**A**) Effect of 5′ and 3′ UTR of TSWV RNA *S* and *sgRNA-NSs* on in vitro translation of the reporter gene with or without 5′ cap. (**B**) In vitro translation of *S* RNA and *sgRNA-NSs* in WGE. (**C**) Effects of deletion mutations of sgRNA-NSs 5′ UTR on the translation of the reporter gene. (**D**) Effects of deletion mutations of *sgRNA-NSs* 3′ UTR on the translation of the reporter gene.

To determine what role the 5′ and 3′ UTRs play in TSWV gRNA and sgRNA translation, in vitro translation assays were performed using wheat germ extract (WGE). The sgRNA-*NSs* 5′ UTR enhanced Fluc translation 5- to 6-fold in the absence of a 5′ cap (Figure 4A), suggesting a cap-independent translation enhancement mechanism. Furthermore, the translational enhancement activity of the *sgRNA-NSs* 5′ UTR was slightly decreased in the presence of the *sgRNA-NSs* 3′ UTR (Figure 4A). It was suggested that *sgRNA-NSs* 3′ UTR could not enhance *F-NSs-5U* translation in the absence of a 5′ cap. Additionally, the *F-Ts-NSs-5U* enhanced the *Fluc* translation to ~20-fold in the presence of the 5′ cap (Figure 4A). Moreover, the *Fluc* fusion constructs *F-Ts-NSs-5U3U* and *F-Ts-S-5U3U* enhanced Fluc translation 44- to 50-fold in the presence of a 5′ cap (Figure 4A). It showed that *Ts-NSs-5U* presents translation enhancement activity in the presence of the 5′ cap, which is synergistically enhanced by the *Ts-NSs-3U*. Taken together, these results suggest that the *sgRNA-NSs* 5′ and 3′ UTRs and cap structure are essential for *Fluc* expression.

To more precisely map the core translational enhancement regions in the *sgRNA-NSs* 5′ and 3′ UTRs, we constructed a series of deletion mutations. The translation of constructs remained at more than 70% of that of the wild-type (wt) *F-R1-5U*. Deletion of 15 nt from the 5′ end (*F-NSs-5U-D1*) resulted in a reduction in translation of <30% of that of F-Ts-NSs-5U (Figure 4C). The deletion of 30 nt from either the 5′ end (*F-NSs-5U-D2*) or the 3′ end (*F-NSs-5U-D3*) resulted in a reduction in translation of >80% of that of *F-Ts-NSs-5U*. Deletion of the A-rich region of the IGR (*F-TS-N-5U3U(Da)*) resulted in a 50% reduction of *F-Ts-NSs-5U3U* translation (Figure 4D), while keeping only the A-rich region of the IGR resulted in a slight enhancement of *Ts-NSs-5U3U* translation. In order to further evaluate the function of the 5′ cap structure in the full-length *gRNA-S* and *sgRNA-NSs*, in vitro synthesized *S* RNA and *sgRNA-NSs* were translated using WGE. The translation was enhanced by 20- to 30-fold in the presence of a 5′ cap, compared to uncapped *gRNA-S* and *sgRNA-NSs* (Figure 4B). It appears that the core translational enhancement region in the *sgRNA-NSs* 5′ UTR is located between positions 1 and 15, that the A-rich region within the 3′ UTR may enhance translation by acting as a poly(A) tail, and that the 5′ cap structure can significantly enhance the efficiency of RNA translation.

### 2.5. The TSWV sgRNA-NSs, but Not RNA S, Is 5′ Capped In Vivo

**Figure 5:**
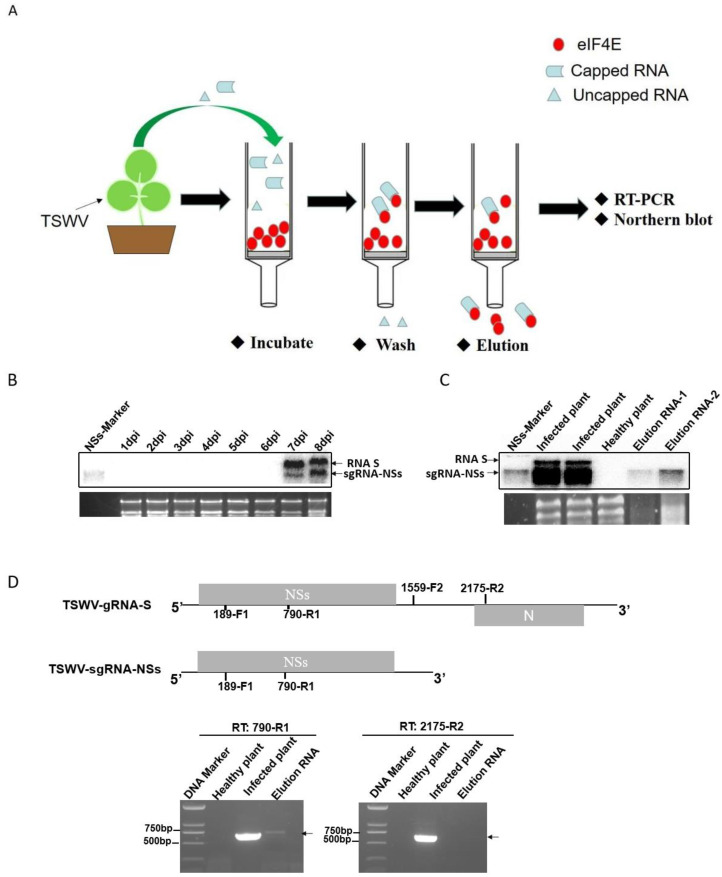
Enrichment of the 5′-capped RNAs due to high affinity for eIF4E. (**A**) 5′-capped RNA enrichment process. (**B**) Northern blot of total RNA isolated from TSWV-infected tobacco (*N. benthamiana*) leaves. The arrows indicate TSWV gRNA and sgRNA. (**C**) Northern blot analysis of the eluted RNA. The arrows indicate TSWV gRNA and sgRNA. (**D**) RT-PCR analysis of the eluted RNA using different primers. The arrow indicates PCR products.

Based on the above data, sgRNA failed to present an advantage in protein expression over the corresponding gRNA when both sgRNA and gRNA possess or lack a 5′ cap. To investigate the reason for the translational advantage of sgRNA relative to corresponding genomic RNAs, the existence or not of 5′ cap in gRNA and sgRNA was analyzed. Similar to other segmented, negative-strand (−) RNA viruses, TSWV transcription utilizes a ‘cap snatching’ mechanism to obtain a 5′ cap structure from host mRNAs. To ascertain whether TSWV gRNA and sgRNA are both capped in vivo, we experimentally enriched the 5′-capped RNA by making use of the affinity of eIF4E for the cap structure (Figure 5A). We introduced TSWV directly into young tobacco leaves through mechanical inoculation and isolated total RNA 7 d later (Figure 5B). To enrich TSWV mRNA, the purified His-eIF4E fusion protein was mixed with the total RNA extracted from TSWV-infected tobacco, and the capped RNA was obtained by co-precipitation. Both northern blot and RT-PCR indicated that only the *NSs* sgRNA could be detected (Figure 5C,D), suggesting that TSWV sgRNA, but not gRNA, is capped in vivo.

## 3. Discussion

### 3.1. Molecular Characteristics of TSWV sgRNAs Superior to gRNAs in Viral Proteins Expression

Although the TSWV gRNAs (*M, S*, and complementary strands) have the ability to encode protein, the two non-structural proteins (NSs and NSm) are expressed from sgRNAs in vivo [33,34]. The TSWV mRNA IGR plays an essential role in sgRNA synthesis, and the IGR contains A- and U-rich sequences which are predicted to fold into a stable hairpin structure [12,32,35]. Previous studies reported that the 3′ ends of the sgRNAs cover the entire hairpin structure, as indicated by both RT-PCR and northern blot hybridization. However, our results indicate that the 3′ ends of sgRNAs do not cross the top of the intergenic hairpin and instead disrupt the base pairing between A and G, resulting in the 3′ UTR of TSWV sgRNA containing only the A-rich sequence. Furthermore, sequence analysis revealed that while the sgRNA 3′ terminal nucleotides and their flanking sequences were conserved, they did not contain a conserved motif (CCGUCG) that is known to play a role in transcriptional termination.

While previous studies have suggested that the IGR plays an important role in translation, the exact role of the sgRNA 3′ UTR conserved sequence remains to be elucidated. In this study, we investigated the role of both *sgRNA-NSs* 5′ UTR and 3′ UTR in the in vitro enhancement of RNA translation. The *sgRNA-NSs* 5′ UTR was found to enhance translational efficiency at least 5-fold, compared to Fluc in the absence of the 5′ cap. The translational efficiency of the *sgRNA-NSs*-5′ UTR was enhanced even more in the presence of the 5′ cap. Moreover, the *sgRNA-NSs* 5′ UTR appears to perform cap-dependent translational enhancement, and its activity is synergistically enhanced by the 3′ UTR, likely due to the A-rich sequence within the hairpin structure acting as a poly(A) tail.

In general, the mechanism by which these viral sgRNAs are efficiently translated is poorly understood [36,37]. Here we have described the cap-independent translational enhancement of TSWV gRNAs and sgRNAs. Both the 5′ and 3′ UTRs were found to significantly enhance translational efficiency in the presence of the 5′ cap. 

It is well known that TSWV gRNA synthesis is initiated by ‘cap snatching’ to gain a 5′ cap structure [38]. However, the process of cap-snatching, and in particular the involvement of host- or virus-encoded endonuclease activity, needs to be investigated further. Furthermore, the literature regarding the cap status of TSWV gRNAs and sgRNAs is ambiguous. For example, a recent report on the *carnation mottle virus* (CarMV) indicated that its gRNAs were capped [33,39]. However, in the current study, we found that TSWV gRNAs are not capped in vivo, which may explain why viral protein biosynthesis relies on sgRNA templates.

### 3.2. Possible Mechanisms of sgRNA Synthesis of Ambisense RNA Viruses

Production of sgRNAs for efficient translation of downstream viral proteins is one of the major strategies adapted for viruses that contain a multicistronic RNA genome [40,41]. For positive-strand RNA viruses, three different modes have been described for the synthesis of sgRNAs. The first mechanism involves internal initiation on a (−)-strand RNA template and requires an internal promoter. The second mechanism makes a prematurely terminated (−)-strand RNA which is used as a template to make the sgRNA. The third mechanism uses discontinuous RNA synthesis while making the (−)-strand RNA templates [42,43,44]. However, most cytoplasmic-replicating negative-strand RNA viruses initiate the synthesis of gRNA and sgRNA by cap snatching [41]. In this study, TSWV contain a segmented single-stranded RNA genome with negative polarity and use an ambisense coding strategy via the production of a set of sgRNAs [29]. Understanding the mechanisms of sgRNA synthesis could shed light on how these RNA viruses replicate.

**Figure 6:**
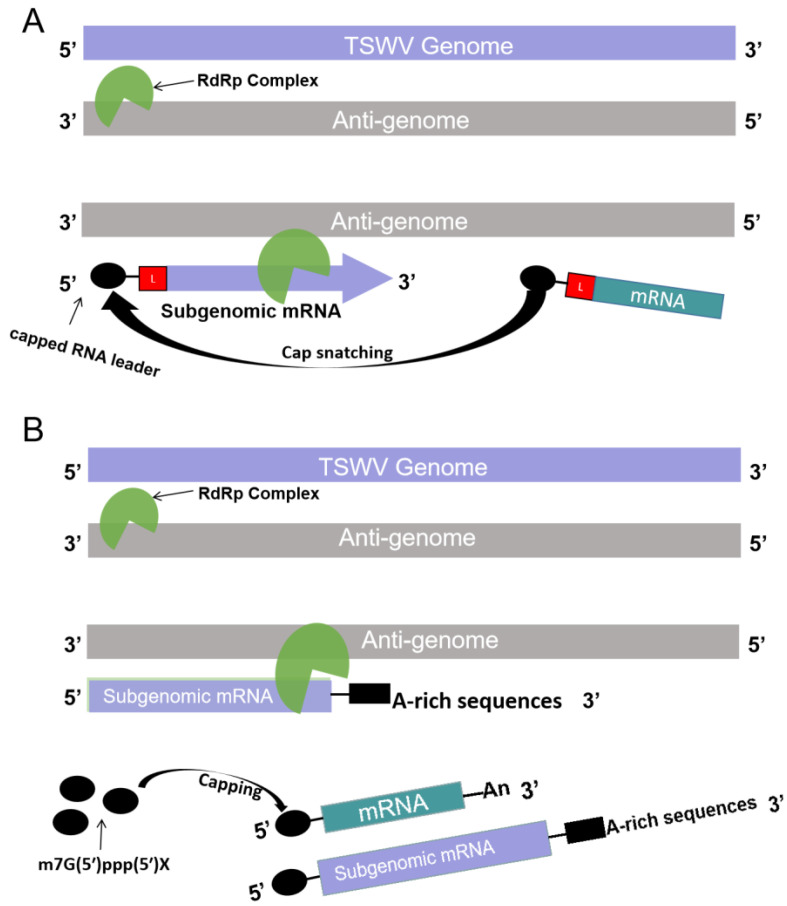
Diagrams of models of TSWV sgRNA generation. (**A**) The viral RNA polymerase cleaves a capped RNA leader from host cellular mRNAs and use these as primers for transcription on the viral genome for sgRNA generation. (**B**) mRNAs and sgRNA are capped by capping enzyme.

For ambisense RNA viruses, the synthesis of sgRNAs was initiated with cap-snatching [45]. Previous studies on bunyavirus TSWV and orthomyxovirus influenza A indicate that the selection and cleavage of host cellular mRNA leaders involve similar criteria for all segmented (−) RNA viruses [33,46,47]. In addition, two models were proposed to explain the acquisition of the 5′ cap structure by TSWV sgRNAs in this study. The first kind of model is based on the snatching of a capped RNA leader from host cellular mRNAs. Specifically, once the TSWV enters the plant cells, full-length genomic-minus strand RNAs were synthesized from the genomic RNA by RNA-dependent RNA polymerase (RdRp) if without a capped RNA leader. But if the viral RdRp cleaves a capped RNA leader from host cellular mRNAs to use these as primers for transcription on the viral genome, that could initiate the synthesis of a sgRNA (Figure 6A). The other model suggested that the step of ‘cap snatching’ occurred during the synthesis and processing of TSWV sgRNAs. Primarily, full-length genomic-minus strand RNAs and sgRNA were synthesized from the genomic RNA by RdRp. Because the molecular structure of gRNAs is similar to the structure of sgRNA, such as both possess an A-rich sequence. So, the eukaryotic mRNA along with viral sgRNA were capped by the host plant due to similar features (Figure 6B). The second mode echoes our findings which have shown that TSWV sgRNAs contain 5′ cap and A-rich sequence tail-like common eukaryotic mRNAs. Moreover, it is the structure of sgRNA that plays an essential role in the translation of TSWV [33]. In all, this study not only further enriched the cap-snatching mechanism of 3′-truncated type of sgRNAs, but also provided a better understanding of the sgRNAs synthesis of ambisense RNA viruses.

## 4. Materials and Methods

### 4.1. Rapid Amplification of cDNA Ends (3′RACE)

3′RACE of the TSWV sgRNA was carried out as previously described [48]. Briefly, total RNA and oligo(A)-linker were ligated using T4 RNA ligase (Takara Bio Inc., Kusatsu, Japan). The RNAs were reverse-transcribed, followed by PCR amplification using anti-linker/TSWV-Q0. The final products were cloned into the pMD18-T vector and sequenced using M13 primers. At least three RT-PCR clones were sequenced to ensure the reliability of the 3′-RACE results. All primers used in this study can be found in Supplementary Materials Table S1.

### 4.2. Plasmid Construction

Plasmids were constructed based on the firefly luciferase (*FLuc)* reporter construct pT7-F-3-UTRsspI vector using PCR amplification, enzyme digestion, and ligation. All plasmid nucleotide sequences were confirmed by DNA sequencing. All FLuc reporter constructs were linearized with NruI/SspI to create the template for RNA preparation.

### 4.3. RNA Preparation

RNA was transcribed in vitro using bacteriophage T7 RNA polymerase (Promega, Madison, WI, USA), according to the manufacturer’s instructions. RNA integrity was estimated by 1.0% agarose gel electrophoresis, and RNA concentration was measured using a NanoDrop spectrophotometer.

### 4.4. In Vitro Luciferase Translation Assays

In vitro luciferase translation assays were performed as previously described [49]. Briefly, 3 pmol of RNA transcripts was used in a 25 μL translation reaction with wheat germ extract (WGE) (Promega), according to the manufacturer’s instructions. Luciferase activity was measured using a luciferase assay reporter system (Promega) and a Modulus Microplate Multimode Reader (Turner BioSystems, Sunnyvale, CA, USA). At least three independent in vitro translation assays were performed for each construct. Standard errors were calculated in Microsoft Excel.

### 4.5. In Vitro Translation of TSWV gRNAs and sgRNAs

TSWV gRNA and sgRNA constructs were linearized with Sma and served as the templates for RNA transcription using T7 RNA polymerase. The 20 μL in vitro translation mixture contained 10 μL WGE (Promega), 0.5 pmol RNA template, 0.8 μL 1 mM amino acids mix (without methionine), 100 mM potassium acetate, and 0.5 μL [5 μCi] 35S-methionine. The translation mixture was incubated at 25 °C for 1 h and then resolved on a 10% sodium dodecyl sulphate-polyacrylamide gel electrophoresis (SDS-PAGE) gel. The gel was dried and subjected to Fujifilm Phosphorimager screening for 3 h. The screen was subsequently scanned by an Amersham Typhoon fluorescent image analyzer. Radioactive band intensity was quantified using ImageQuant TL 8.1 (GE Lifesciences, Piscataway, NJ, USA). All experiments were repeated at least three times.

### 4.6. eIF4E Expression and Purification

The wheat eIF4E coding sequence (KX467331) was amplified by reverse transcription PCR (RT-PCR) and cloned into the BamH I and Sal I restriction sites of a pEHisTEV vector. The positive plasmid containing eIF4E (KX467331) was transformed into Escherichia coli ‘Rosetta’. eIF4E protein expression was induced with 0.5 M isopropyl-Dthiogalactoside (IPTG) at 37 °C for 5 h. Samples were separated by SDS-PAGE. The gel bands containing the 24 kDa eIF4E protein were cut and placed into dialysis bags. After 2 h of electrophoresis in SDS-PAGE buffer, the gel in the dialysis bags was removed and the dialysis bags were dialyzed for 24 h followed by split charging of the eIF4E proteins.

### 4.7. RT-PCR Detection of TSWV gRNAs and sgRNAs

Total RNA was extracted from tobacco (*N. benthamiana*) leaves using Trizol reagent (TransGen Biotech Inc., Beijing, China), and reverse-transcribed using M-MLV reverse transcriptase and oligonucleotides corresponding to TSWV. Then, PCR amplification was performed using Taq DNA polymerase and pairs of oligonucleotides (Supplementary Materials Table S1). To detect TSWV, two pairs of oligonucleotides, TSWV-F/TSWV-3263-R and TSWV-F/TSWV--R, were designed for the RT-PCR assay.

### 4.8. Northern Blot

Total RNA was extracted from systemic non-inoculated leaves 7 days post infiltration (dpi) after agroinfiltration, and northern blotting was performed as previously described. cDNA probes were labeled using specific primers to detect TSWV RNA S and sgRNA-NSs (Supplementary Materials Table S1). For northern blotting, RNA was subjected to electrophoresis through 2% agarose gels and transferred to a charged nylon membrane by capillary action in 4 × SSC (1 × SSC is 0.15 M NaCl plus 0.015 M sodium citrate) buffer. After UV cross-linking, membranes were hybridized with the mixture of three [α-32]dATP-labeled DNA oligonucleotides complementary to the sequence of TSWV.

## Data Availability

Not applicable.

## Supplementary Materials

The supporting information can be downloaded at: https://www.mdpi.com/article/10.3390/ijms232315074/s1.
