# Supplementary material for: Molecular Characteristics of Subgenomic RNAs and the Cap-Dependent Translational Advantage Relative to Corresponding Genomic RNAs of Tomato spotted wilt virus
Source: Int J Mol Sci. 2022 Dec 1;23(23):15074. doi: 10.3390/ijms232315074 (PMC9741439; doi:10.3390/ijms232315074)
Supplement: Supplementary file 1 [file ijms-23-15074-s001.zip › ijms-2005295-supplementary.pdf]

Table S1 Primers in this study

Primer name	Primer sequence (5'→3')	Uses for the primer
Qa-F	5′-AAAAAAAAAAAAAAAAAGCTTGAGCTCGAGTCCTCGTCACTCTGCTCACTGG-3′	3′RACE of the TSWV sgRNA
Q0	5′-CCAGTGAGCAGAGTGACG-3′	3′RACE of the TSWV sgRNA
Q1	5′-GAGGACTCGAGCTCAAGC-3′	3′RACE of the TSWV sgRNA
TS-S-1185-F	5′-TGAACAGCTATGAGCTAACTCC-3′	3′RACE of the TSWV sgRNA
TS-S-VC-702-F	5′-ATGACACCAGGGAAGCCTTAG-3′	3′RACE of the TSWV sgRNA
TS-NSs-Bam5U-D1-F	5′-CAGGATCCTAATTTTATTCTTAATCAAACCTCA-3′	F-Ts-NSs-5U-D1 reporter construct
TS-NSs-Bam5U-D2-F	5′-CAGGATCCAGAGCAATTGTGTCAAAAATCACAATACTG-3′	F-Ts-NSs-5U-D2 reporter construct
TS-NSs-Sm5U-D2-R	5′-CACCCGGGTATGGTTATTGGTACTGTGTTCTTA-3′	F-Ts-NSs-5U-D2 reporter construct
TS-NSs-Bam5U-D3-F	5′-CAGGATCCAGAGCAATTGTGTCATAATTT-3′	F-Ts-NSs-5U-D3 reporter construct
TS-NSs-Sm5U-D3-R	5′-CACCCGGGGTATTGTGATTTTCTAAGTGAGGT-3′	F-Ts-NSs-5U-D3 reporter construct
Ts-BamHI-S-5U-F	5′-CAGGATCCAGAGCAATTGTGTCATAATTTTATTCTTAATCAAAC-3′	F-Ts-S-5U, reporter construct
Ts-SmaI-S-5U-R<br>Ts-SmaI-NSs-5U-R	5′-CACCCGGGTATGGTTATTGGTACTGTGTTCTTATTACAGT-3′	F-Ts-S-5U-3U, reporter construct
Ts-Nru1-S-3U-F	5′-GCTCGCGAGATGATCGTAGAAGTTGTTATATGCT-3′	F-Ts-S-5U-3U, reporter construct
Ts-Ssp1-S-3U-R	5′-GCAATATTAGAGCAATTGTGTCAATTTTATTCAAACCTTAACAC-3′	
Ts-Nru1-NSs-3U-F	5′-GCTCGCGATCTTGCCGTGTCCAGCTTTT-3′	F-Ts-S-5U-3U, reporter construct
Ts-Ssp1-NSs-3U-R	5′-GCAATATTGTTGTTTTTATTTCATTTTTTGATTTTTGTTTGATTTTGT-3′	
Ts-BamHI-T7-S-5U-F:	5′-CAGGATCCTAATACGACTCACTATAGGAGAGCAATTGT	In vitro transcription for TSWV gRNA
Ts-BamHI-T7-NSs-5U-F	5′-GTCATAATTTTATTCTTAATCAAAC-3′	In vitro transcription for TSWV sgRNAs NSs
Ts-SmaI-S-3U-R	5′-CACCCGGGAGAGCAATTGTGTCAATTTTATTCAAACCTTAACAC-3′	In vitro transcription for TSWV gRNA S
Ts-SmaI-NSs-3U-R	5′-CACCCGGGGTTGTTTTTATTTCATTTTTTGATTTTTGTTTGATTTTGT-3′	In vitro transcription for TSWV sgRNAs NSs
TS-NSS-NT-630-R	5′-CCCAAAGCTTGATTGTAGCACATC-3′	cDNA probe
TS-S-189-F	5′-AACTTGGTACTGGTTCTCCAC-3′	RT-PCR detection of TSWV gRNAs and sgRNAs
TS-S-790-R	5-CTGAGAGTTAGGCATGATGTTG-3	RT-PCR detection of TSWV gRNAs and sgRNAs
TS-S-1559-F	5-TCTGTTTTGTCATTTCTTTTGAATTTCT-3	RT-PCR detection of TSWV gRNAs
TS-S-2175-R	5-TGCTGCTATACTTAGCTCCAGC-3	RT-PCR detection of TSWV gRNAs

TS-NSS-NT-412-F	5′-CGGAGTGAAACATCAAGGTCATTT-3′	cDNA probe
TS-NSS-NT-630-R	5′-CCCAAAGCTTGATTGTAGCACATC-3′	cDNA probe
